# lncRNA H19 promotes matrix mineralization through up-regulating IGF1 by sponging miR-185-5p in osteoblasts

**DOI:** 10.1186/s12860-019-0230-3

**Published:** 2019-11-12

**Authors:** Yuan Wu, Yu Jiang, Qiang Liu, Cui-Zhong Liu

**Affiliations:** 1 Department of General Practice, Hunan Provincial People’s Hospital, No.61, Jiefang West Road, Changsha, 410006 Hunan Province People’s Republic of China; 2 Hunan Provincial People’s Hospital, Institute of Emergency Medicine, Hunan Provincial Key Laboratory of Emergency and Critical Care Metabonomics, Changsha, 410006 People’s Republic of China; 3 Department of Hepatobiliary Surgery, Hunan Provincial Cancer Hospital, The Affiliated Cancer Hospital of Xiangya School of Medicine, Central South University, Changsha, 410013 People’s Republic of China

**Keywords:** lncRNA H19, miR-185-5p, IGF1, Osteoblasts, Mineralization

## Abstract

**Background:**

Matrix mineralization is a key stage in bone formation involving in many bone-specific genes and signaling pathways. Emerging evidence indicate that long non-coding RNA (lncRNA) and microRNAs (miRNAs) play crucial roles in regulating the mineralization process of osteoblasts. This study aims to characterize the function and mechanism of lncRNA H19/miR-185-5p/IGF1 axis in modulating matrix mineralization of osteoblasts.

**Results:**

H19 and IGF1 were highly expressed while miR-185-5p was lowly expressed in mineralized cells. Knocking down H19 inhibited matrix mineralization of osteoblasts, yet miR-185-5p had opposite effects. Moreover, H19 directly targeted miR-185-5p, whereas miR-185-5p repressed IGF1 expression. Meanwhile, miR-185-5p inhibition compensated the suppression of the matrix mineralization in osteoblasts by H19 knockdown.

**Conclusions:**

The findings of this study showed that lncRNA H19 was upregulated in mineralized osteoblasts and promoted matrix mineralization through miR-185-5p/IGF1 axis in osteoblasts for the first time. This study may provide a new perspective for the diagnosis and treatment of diseases related to bone metabolism.

## Background

Bone metabolism is a continual cycle of bone growth and resorption that is carefully orchestrated by the dynamic relationship between osteoclasts, osteoblasts and an array of hormonal and regulatory influences [1, 2]. The steps of osteogenic proliferation, differentiation, and bone homeostasis are controlled by various markers and signaling pathways [3]. Bone remodeling is delicately regulated by both the number and activity of osteoblasts, which are bone-forming cells, and osteoclasts, which are bone-degrading cells [4]. The process of osteoblastogenesis can be divided into steps comprising proliferation, extracellular matrix development and maturation, mineralization, and apoptosis, which are controlled by a well-defined genetic program [5, 6].

The pathological state of various bone metabolism-related diseases such as osteoporosis is closely related to mineralization of osteoblasts [7]. It is well known that matrix mineralization plays a critical role in bone formation [8]. At the end of osteogenic differentiation, mineralized nodules formed in osteoblasts [9]. Differential expression of bone-related genes and signaling molecules may be involved in the regulation of osteoblasts mineralization [10].

Long noncoding RNAs (lncRNAs) are RNAs longer than 200 nucleotides in length participating in a variety of cellular processes as potential biological regulators, such as gene expression, translation regulation, and signal transduction [11]. Recently, they were also reported to participate in the regulation of osteogenic activity [12]. H19 was the first lncRNA ever discovered, and has many diverse biological functions, participating in the regulation of cell proliferation, differentiation and metabolism [13]. Increasing evidences indicated that H19 was involved in bone regeneration process. It could promote osteogenic differentiation by activating Wnt signaling [14] and it could also mediate mechanical tension-induced osteogenesis via focal adhesion kinase (FAK) [15]. However, it has not been fully elucidated the function and mechanism in mineralization of osteoblasts. Therefore, it is of great significance to study the regulatory role of lncRNA H19 and molecular mechanisms regulating the mineralization process of osteoblasts for better understanding bone formation and bone diseases.

Recently, competitive endogenous RNA (ceRNA) has been reported as a new underlying mechanism of lncRNA, which lncRNAs act as microRNAs (miRNAs) sponges and regulate the expression of downstream target mRNA of those miRNAs [15]. MiRNAs are approximately 22 nucleotides in length and act as gene expression regulators involved in the regulation of multiple life processes [16]. Several miRNAs have been identified to regulate cancer growth and stemness, osteogenetic process [17, 18]. miR-185-5p was reported to be a bone specific circulating miRNAs and participated in regulation of cell growth, proliferation and apoptosis [19] and miR-185-5p over-expression inhibited amelogenesis and osteogenesis [20]. While, it remains to be verified the molecular mechanisms of miR-185-5p in regulating the process of osteogenic mineralization. Insulin-like growth factor 1 (IGF1), an important factor in growth and development, played essential roles in bone matrix mineralization [21, 22]. IGF1 up-regulated Collagen Type I Alpha 2 Chain (COL1A2) protein expression and alkaline phosphatase (ALP) activity in the primary osteoblasts and IGF1 promoted osteogenic differentiation and mineralization in vitro [23].

In this study, we showed that lncRNA H19 was upregulated in mineralized osteoblasts and promoted matrix mineralization of osteoblasts through miR-185-5p/IGF1 axis suggesting a regulatory role for H19 and miR-185-5p in the pathophysiological process leading to bone formation in skeletal disorders for the first time. This study may provide a new perspective for the diagnosis and treatment of diseases related to bone metabolism.

## Results

### Differential expression of H19, miR-185-5p and IGF1 in mineralized osteoblasts

**Fig. 1:**
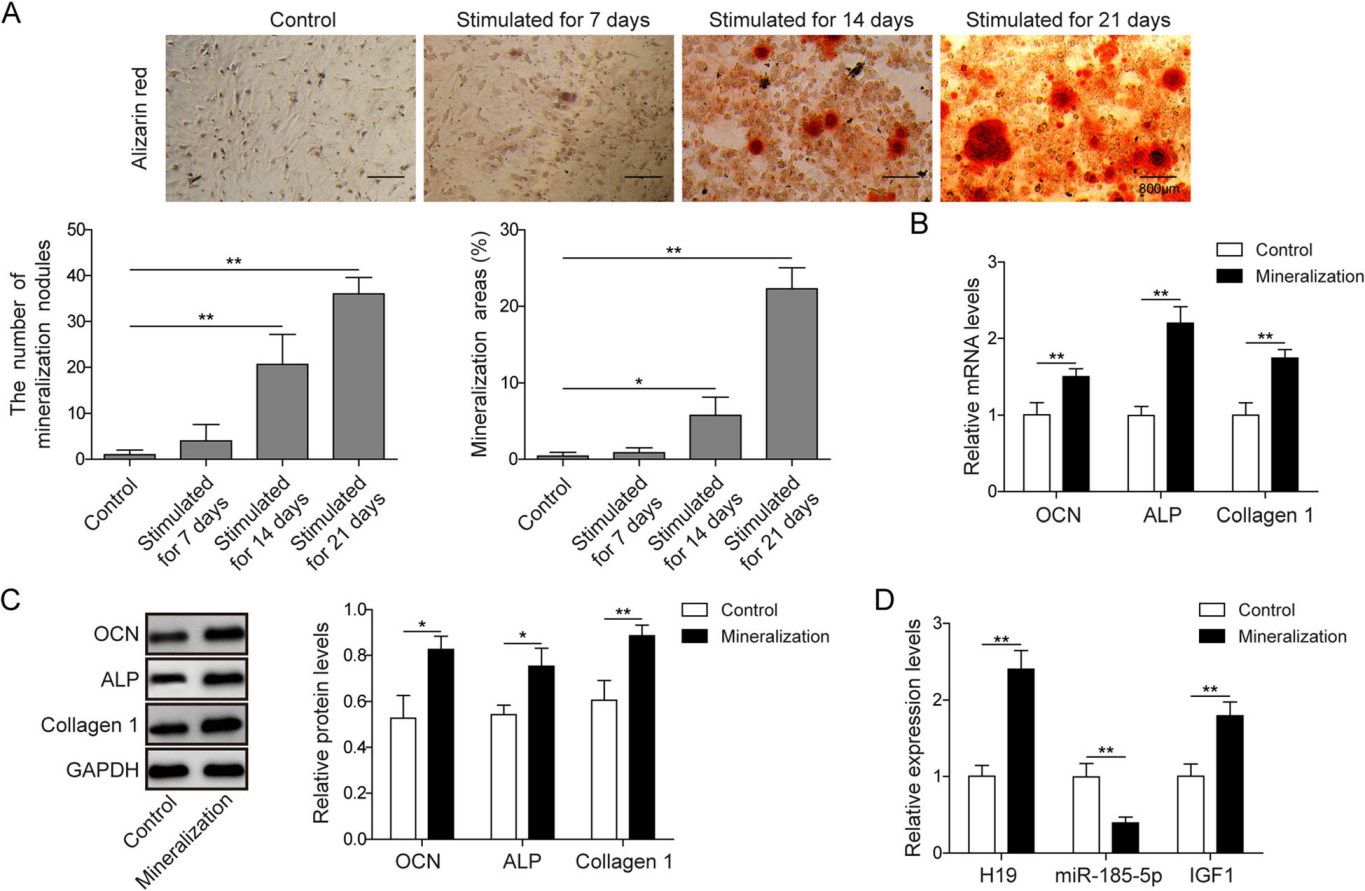
The expression of lncRNA H19 and miR-185-5p in mineralized osteoblasts. **a** The results of Alizarin Red staining, mineralization nodules and ralative areas at 7, 14 and 21 day after induced mineralization of MC3T3-E1 cells. Scale bars, 800 μm. **b**, **c** The mRNA expression of OCN, ALP and Collagen I in control and mineralized MC3T3-E1 cells were assessed by qPCR and western blot on day 7. GAPDH was used for normalization. **d** The expression of H19, IGF1 and miR-185-5p in control and mineralized MC3T3-E1 cells were detected by qPCR. GAPDH or U6 was used for normalization, respectively. The asterisks show difference significant as ** *p* < 0.01, * *p* < 0.05

In order to investigate the expression of bone-specific genes in osteoblasts mineralization, ascorbic acid + β-glycerophosphate was used to induce osteoblasts mineralization. As shown in Fig. 1a, a remarkable increase in Alizarin Red staining, mineralization nodules and ralative areas were observed from day 7 to day 21 after induction of mineralization in MC3T3-E1 cells. Then osteogenic gene expression was assessed by qPCR and western blot. The markers of osteoblastic mineralization OCN, ALP and Collagen I were all up-regulated in mineralized group (Fig. 1b-c). Furthermore, we measured H19, miR-185-5p and IGF1 levels in control and mineralization groups by qPCR. H19 and IGF1 were both up-regulated, while miR-185-5p was down-regulated in the mineralization of cells (Fig. 1d).

### H19 knockdown inhibits matrix mineralization of osteoblasts

**Fig. 2:**
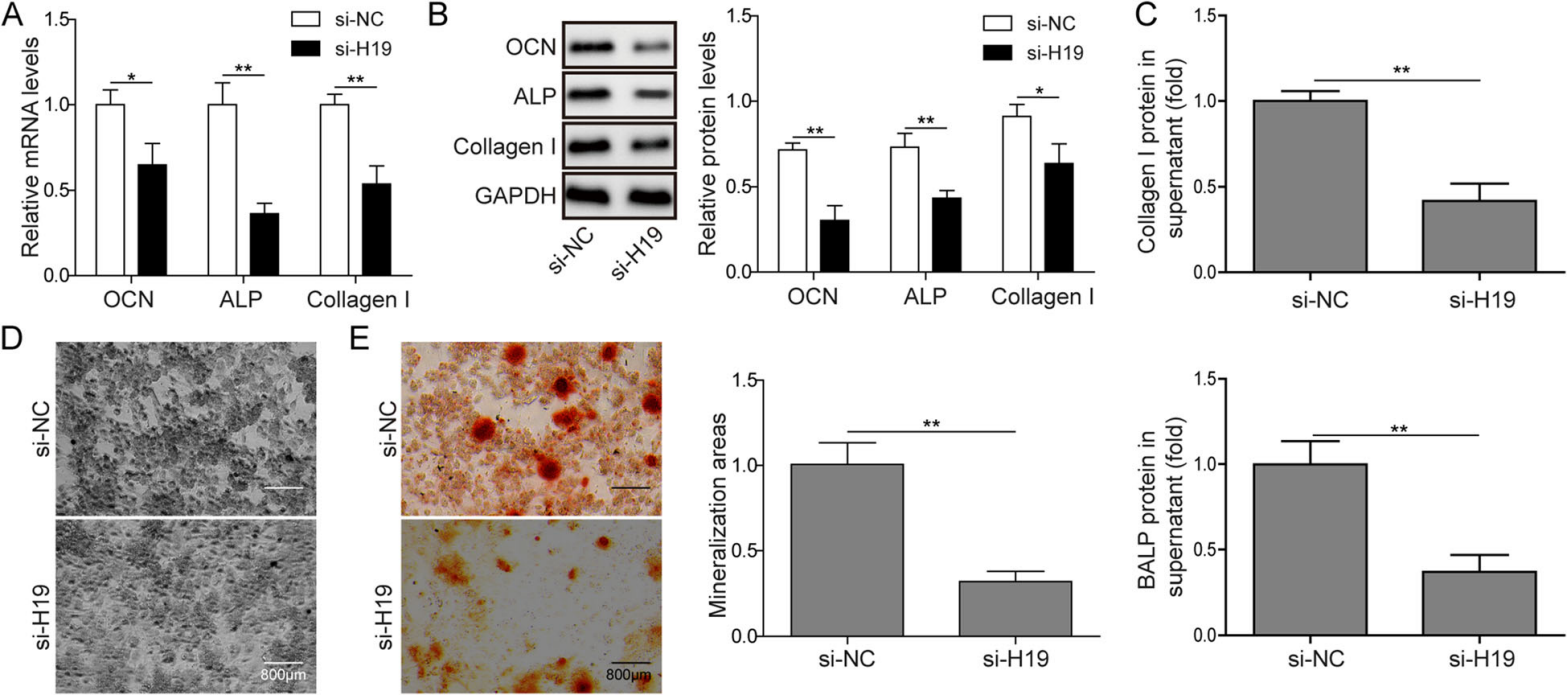
H19 knockdown inhibits matrix mineralization of osteoblasts. **a**, **b** The mRNA expression of OCN, ALP and Collagen I in control and H19 knockdown group of MC3T3-E1 cells were analysed by qPCR and western blot. GAPDH was used for normalization. **c** ELISA analysis of the amount of Collagen I and BALP protein in the supernatant of MC3T3-E1 cells after treatment with NC and H19 siRNA. **d** Representative images of ALP staining of MC3T3-E1 cells on day 7 after treatment with NC and H19 siRNA. Scale bars, 800 μm. **e** Staining of calcium deposition by Alizarin Red in MC3T3-E1 cells on day 14 treated with si-NC and si-H19. Scale bars, 800 μm. The asterisks show difference significant as ** *p* < 0.01, * *p* < 0.05

**Fig. 3:**
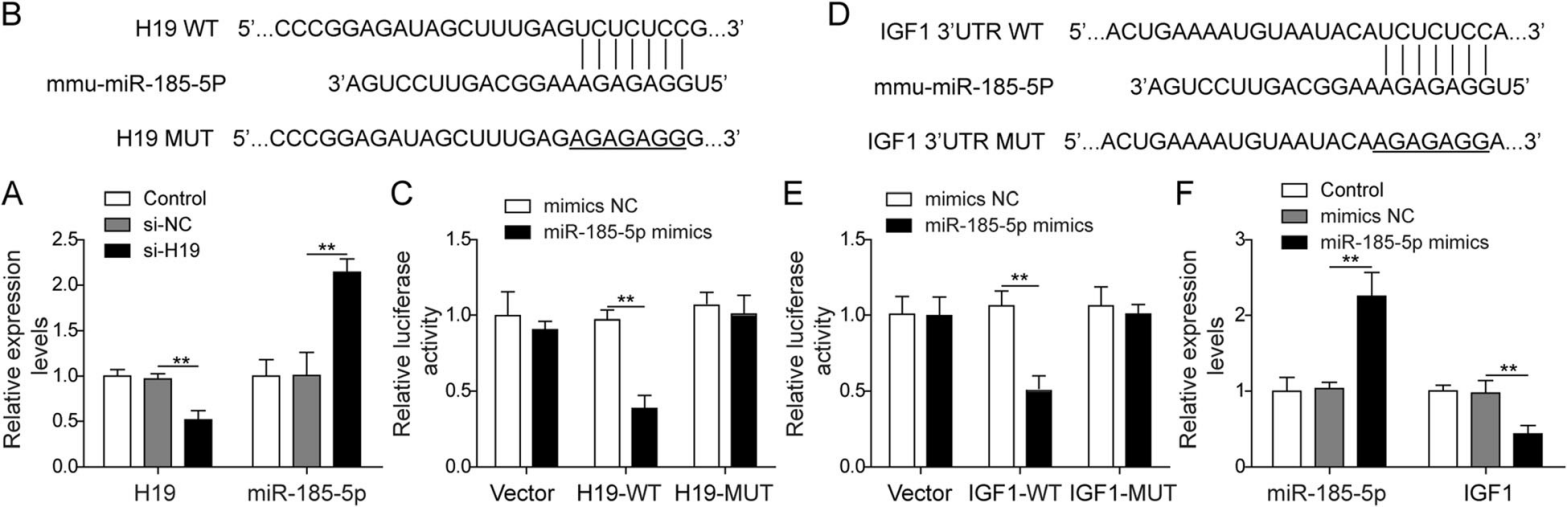
H19 acts as a ceRNA by sponging miR-185-5p and regulated IGF1 expression indirectly. **a** The expression level of miR-185-5p in H19 knockdown treated MC3T3-E1 cells by qPCR. U6 was used for normalization. **b** The predicted binding site between the lncRNA H19 and miR-185-5p by starBase. *Mus musculus*: mmu. **c** The luciferase activity of the H19-WT and H19-MUT in cells treated with miR-185-5p mimics or miR-NC. **d** The predicted binding site between the miR-185-5p and IGF1 by starBase. **e** The luciferase activity of the IGF1-WT and IGF1-MUT in cells treated with miR-185-5p mimics or miR-NC. **f** The expression level of IGF1 in miR-185-5p mimics treated cells by qPCR. The asterisks show difference significant as ** *p* < 0.01, * *p* < 0.05

To investigate the role of H19 in regulating osteoblastic mineralization, mouse pre-osteoblast MC3T3-E1 cells were treated with either si-H19 or si-NC. Intracellular H19 levels were substantially downregulated about 50% by si-H19 treatment (Fig. 3a). Further, mRNA and protein levels of OCN, ALP and Collagen I, three markers of osteoblastic mineralization, were all down-regulated by H19 knockdown compared to treatment with negative control (Fig. 2a-b). Functionally, the supernatant protein concentrations of Collagen I and BALP were substantially lower in the H19 knockdown group (Fig. 2c). Consistent with the changes in the supernatant BALP concentrations, H19 knockdown also weakened ALP^+^ staining (Fig. 2d). In addition, less mineral deposition was found in H19 knockdown cells than in control group (Fig. 2e). All these results indicated H19 knockdown could restrain matrix mineralization of osteoblasts.

### H19 acts as a ceRNA by sponging miR-185-5p and regulated IGF1 expression indirectly

In order to further explore the molecular mechanism of H19 regulating osteoblasts mineralization, we predicted and validated its downstream target. By starBase, a bioinformatics software, we identified predicted H19 binding miRNAs. Among them, miR-185-5p was reported to participate in modulation of osteogenesis [20]. We hypothesized that it may be a target of H19, which needs further validation. First, miR-185-5p was measured by qPCR and was significantly up-regulated about 50% by H19 knockdown or miR-185-5p over-expression (Fig. 3a, f). Then, we predicted the binding site of miR-185-5p and H19 (Fig. 3b). Luciferase results showed that miR-185-5p mimics remarkably inhibited the luciferase activity of the H19-WT, while no effects on the H19-MUT and pGL3-control vector (Fig. 3c), indicating that H19 could directly sponge miR-185-5p. Similarly, IGF1 was a direct target of miR-185-5p and miR-185-5p mimics significantly suppressed the expression of IGF1 (Fig. 3d-f). Taken together, H19 may act as a ceRNA by sponging miR-185-5p and indirectly regulated IGF1 expression.

### MiR-185-5p inhibits matrix mineralization of osteoblasts

**Fig. 4:**
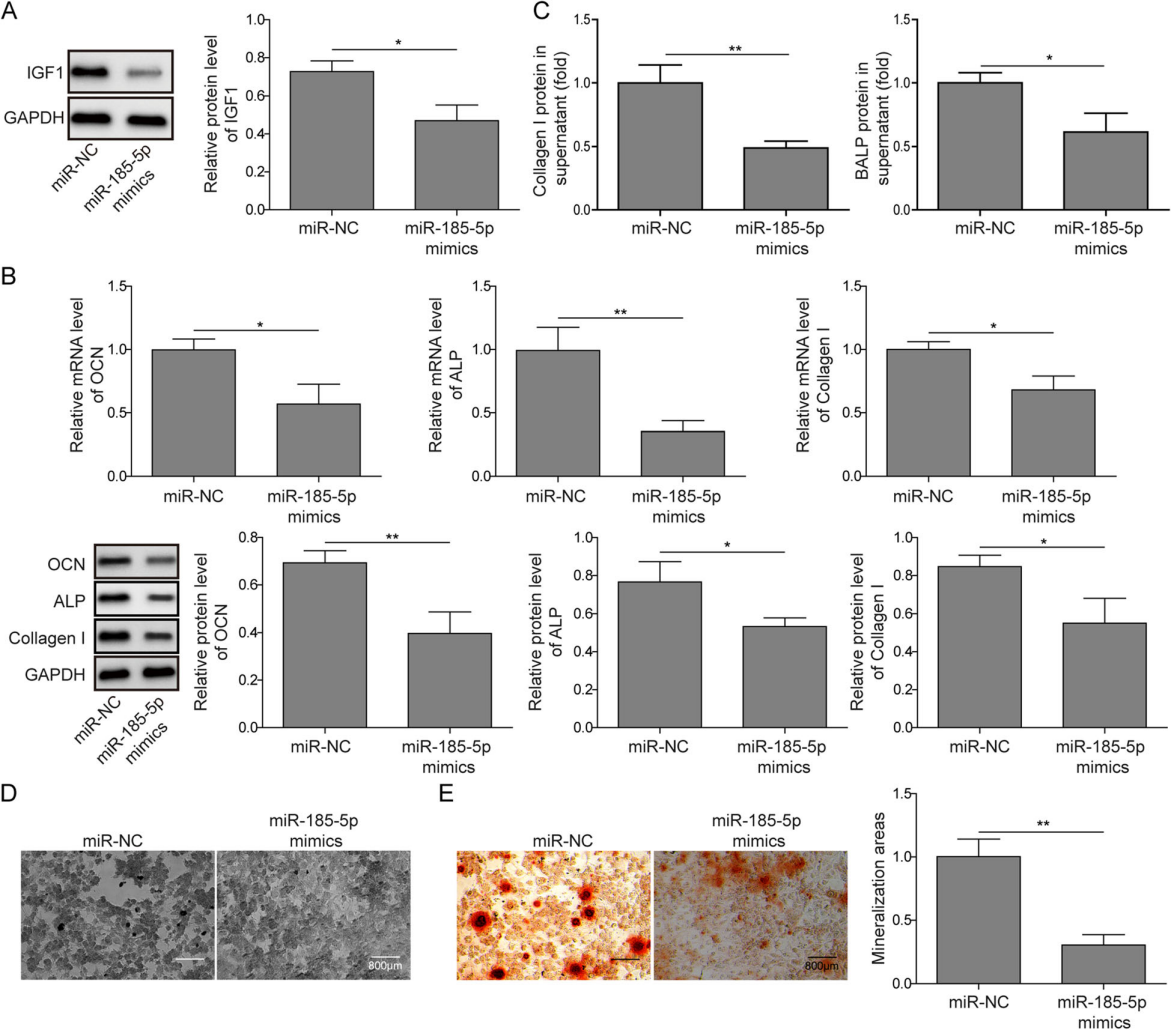
miR-185-5p overexpression inhibits matrix mineralization of osteoblasts. **a** The expression of IGF1 in MC3T3-E1 cells transfected with miR-185-5p mimics and miR-NC by western blot. GAPDH was used for normalization. **b** The mRNA expression of OCN, ALP and Collagen I in in MC3T3-E1 cells transfected with miR-185-5p and NC by qPCR and western blot. GAPDH was used for normalization. **c** ELISA analysis of the amount of Collagen I and BALP protein in the supernatant of MC3T3-E1 cells. **d** Representative images of ALP staining of MC3T3-E1 cells after transfection. Scale bars, 800 μm. **e** Staining of calcium deposition by Alizarin Red in MC3T3-E1 cells after treatment with miR-NC and miR-185-5p mimics. Scale bars, 800 μm. The asterisks show difference significant as ** *p* < 0.01, * *p* < 0.05

IGF1 was reported to play an essential role in bone matrix mineralization. Therefore, the expression of IGF1 was investigated in miR-185-5p overexpression group. As shown in Fig. 4a, miR-185-5p overexpression visibly decreased IGF1 protein level. While, the upregulation of OCN, ALP and Collagen I in cells stimulated by mineralization induction medium were all inhibited by miR-185-5p overexpression (Fig. 4b). Furthermore, the production of Collagen I and BALP in cell supernatant were substantially lower mediated by the miR-185-5p mimics treatment (Fig. 4c). We consistently found that miR-185-5p overexpression reduced ALP^+^ staining and mineral deposition (Fig. 4d-e), indicating miR-185-5p could suppress matrix mineralization in osteoblasts.

### H19 promotes matrix mineralization through miR-185-5p/IGF1 axis in osteoblasts

**Fig. 5:**
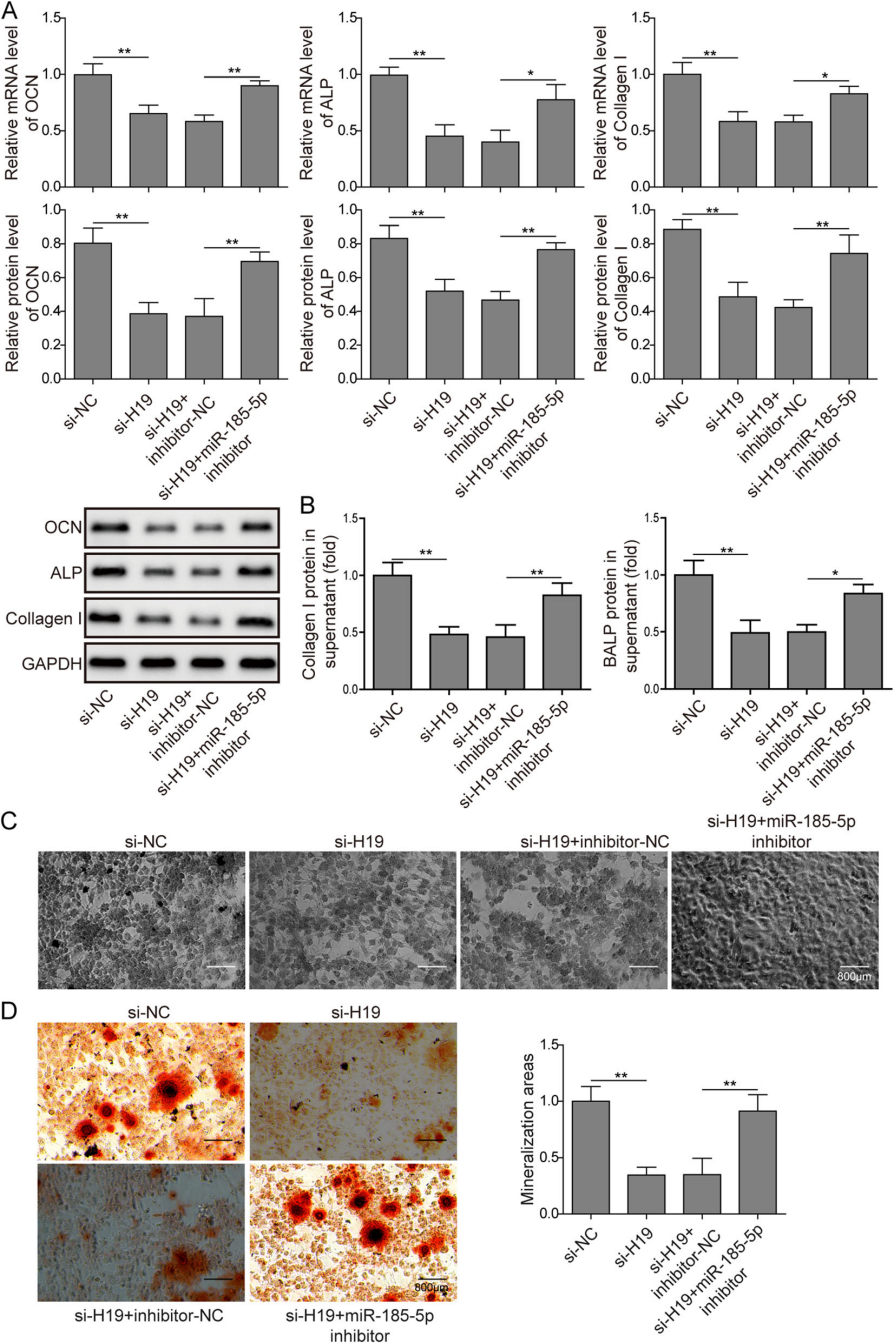
miR-185-5p inhibition rescues the inhibition of matrix mineralization in osteoblasts by H19 knockdown. **a** The mRNA and protein expression of OCN, ALP and Collagen I in H19 knocked-down MC3T3-E1 cells transfected with miR-185-5p inhibitor and NC by qPCR and western blot. GAPDH was used for normalization. **b** ELISA analysis of the amount of Collagen I and BALP protein in the supernatant of in H19 knocked-down MC3T3-E1 cells transfected with miR-185-5p inhibitor and NC. **c** Representative images of ALP staining of transfected MC3T3-E1 cells. Scale bars, 800 μm. **d** Staining of calcium deposition by Alizarin Red in MC3T3-E1 cells transfected with si-H19, miR-185-5p inhibitor and NC. Scale bars, 800 μm. The asterisks show difference significant as ** *p* < 0.01, * *p* < 0.05

To investigate whether H19 regulates matrix mineralization through miR-185-5p/IGF1 axis, miR-185-5p inhibitor and NC were transfected in H19 knocked-down MC3T3-E1 cells and the mRNA and protein expression of OCN, ALP and Collagen I were detected by qPCR and western blot. As shown in Fig. 5a, they were all down-regulated by H19 knocked-down, while miR-185-5p inhibitor could rescue this effect. Functionally, the supression of Collagen I and BALP in the H19 knockdown cells supernatant were partially repaired by miR-185-5p inhibitor (Fig. 5b). Consistent with the changes in the supernatant BALP concentrations, miR-185-5p restraint could rescue the ALP^+^ staining decreased by H19 knockdown (Fig. 5c). Additionally, the mineral deposition in above described groups presented the same variation tendency (Fig. 5d). Collectively, these results indicated that H19 regulates matrix mineralization of osteoblasts through miR-185-5p/IGF1 axis.

## Discussion

LncRNAs play an important role in metabolism of bone, and participate in development and conversion of bone metabolism especially in the regulation of osteogenic differentiation [24]. LncRNA metastasis-associated lung adenocarcinoma transcript 1 (MALAT1) could promote osteogenic differentiation by targeting miR-204 [12]. HOX transcript antisense RNA (HOTAIR) inhibited mineralization in osteoblastic osteosarcoma cells by epigenetically repressing ALPL [25]. Moreover, H19 promoted osteogenic differentiation via Wnt/β-catenin pathway by sponging miR-141 and miR-22 [14]. However, there is no more report of lncRNA H19 in regulation of osteoblasts activity and matrix mineralization. Here, to our knowledge, we reported the regulatory role and molecular mechanisms that H19 played in osteoblasts activity and matrix mineralization.

Studies showed that lncRNA-miRNA-mRNA network plays an important role in the regulation of cell function, including bone metabolism process. lncRNA MEG3 (maternally expressed gene 3) could enhance the expression matrix mineralization-related proteins (ALP, OCN and Collagen I) via the miR-27a-3p/IGF1 signaling pathway [26]. And lncRNA PCAT1 (prostate cancer-associated ncRNA transcript 1)/miR-145-5p/Toll-like receptor 4 (TLR4) signal axis also functions in osteogenic differentiation process [27]. Furthermore, H19 was reported to mediate osteogenesis by sponging miR-138, which is in agreement with the results of our study [15]. In this study, we found that H19 suppression showed inactivation of mineralization related genes such as ALP, OCN and Collagen I, indicating it play a critical role in regulation of matrix mineralization in osteoblasts. Moreover, lncRNA H19 modulated miR-185-5p levels through its function as a miRNA sponge to trap miR-185-5p, therefore regulated the expression of IGF1. And a study have reported that miR-185-5p was associated with osteogenic differentiation through Dlx2 repression [20], which is consistent with our findings. According to the recovery experiment, as the downstream mediator of H19, miRNA-185-5p participates in regulating the expression of mineralization related genes. IGF1, a direct target of miRNA-185-5p, is evolutionarily conserved hormonal signaling molecule, influencing a wide array of physiological functions including bone metabolism [23]. It has been demonstrated that IGF1 promoted osteogenic differentiation during osteoblasts maturation and stimulated osteoblasts proliferation [23]. In the current study, we found that miR-185-5p negatively regulated IGF1 expression, and subsequently facilitated ALP activity and mineralization in MC3T3-E1 cells. Taken together, H19-miR-185-5p-IGF1 regulatory network may exist in mineralization process of osteoblasts and H19 works through ceRNA mechanism. Extracellular matrix mineralization is an important stage of osteoblasts in bone formation. These findings also give insight into the association of H19/miR-185-5p/IGF1 signal axis and osteogenesis, even bone diseases.

Because the development of MC3T3-E1 cells, including ALP activity and matrix mineralization, is similar to bone formation in vivo, thus, this cell line is often used to study bone metabolism related diseases in vitro [28]. Although we have preliminarily explored the molecular mechanism of H19 regulating the matrix mineralization of osteoblasts in vitro, further in vivo experiments are needed to verify the feasibility of H19 as a potential therapeutic target for skeletal diseases in future studies.

## Conclusions

**Fig. 6:**
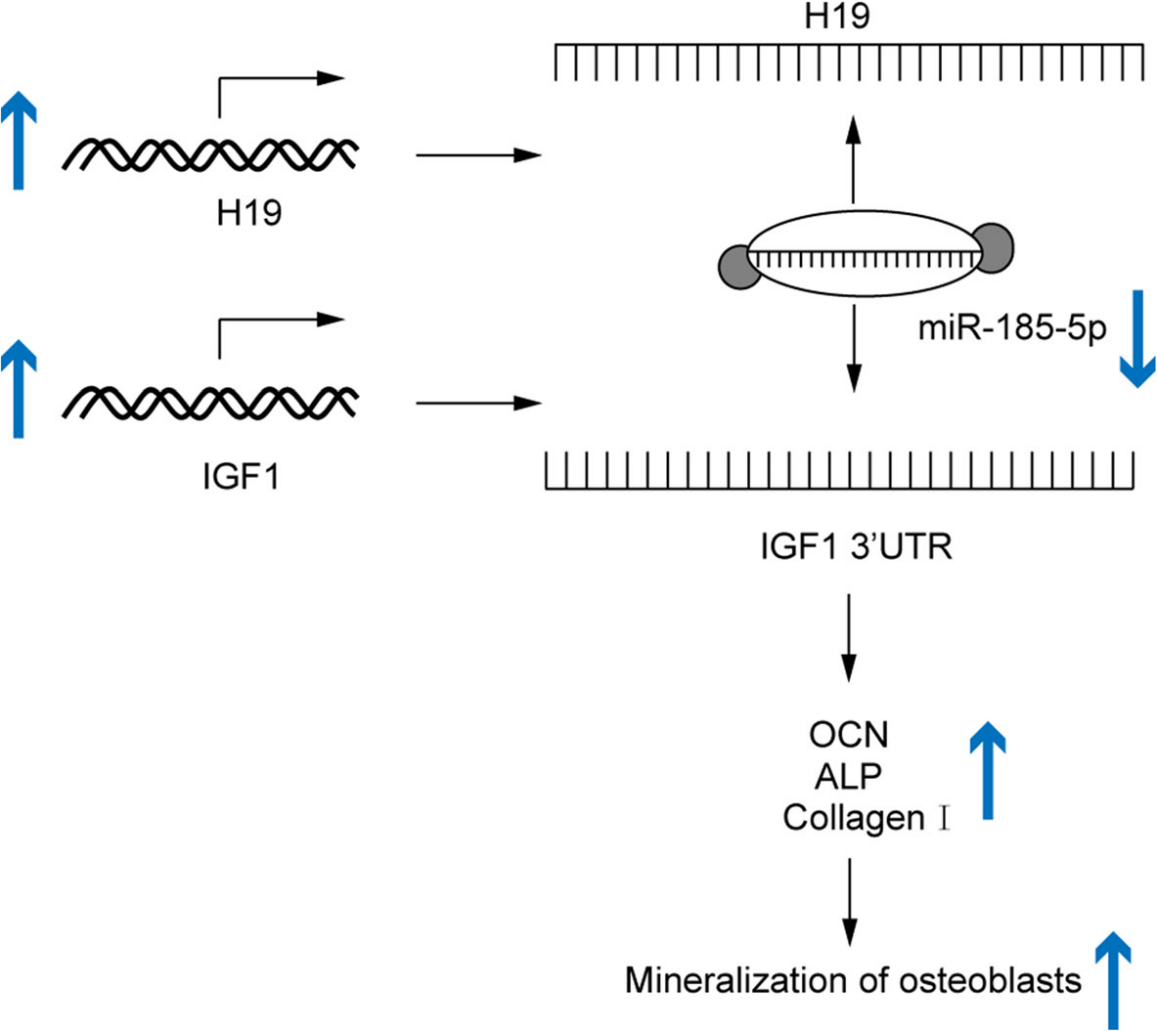
Schematic diagram of proposed mechanism. lncRNA H19 promoted matrix mineralization of osteoblasts through miR-185-5p/IGF1 axis. In mineralization condition, H19 is up-regulated and sponge miR-185-5p. The decreased in miR-185-5p availability, increases the level and translation of IGF1 mRNA, which in turn up-regulates the expression of OCN, ALP and Col1agen I involved in bone matrix mineralization. By contrast, when miR-185-5p is up-regulated, because H19 is decreased, the level of IGF1 is diminished and subsequent inhibition of mineralization, with arrows indicating the process

We show that lncRNA H19 was upregulated in mineralized osteoblasts and promoted matrix mineralization of osteoblasts through miR-185-5p/IGF1 axis (Fig. 6). Our results suggest a regulatory role for H19 and miR-185-5p in the pathophysiological process leading to bone formation in skeletal disorders. These findings may provide a better understanding of the biological effects of lncRNAs on bone formation in vitro and provide a new perspective for the diagnosis and treatment of diseases related to bone metabolism.

## Methods

### Cell culture

MC3T3-E1 cells were obtained from the Chinese Academy of Sciences Cell Bank (Shanghai, China) and grown in α-minimum essential medium (α-MEM; Invitrogen, USA) supplemented with 10% fetal bovine serum (FBS; Invitrogen, USA), 1% penicillin (100 U/ml, GIBCO, USA), streptomycin (100 μg/ml streptomycin; GIBCO, USA), in a humidified atmosphere of 5% CO_2_ and 95% air at 37 °C.

### Mineralization induced by β-glycerophosphate and ascorbic acid

MC3T3-E1 cells were divide into 2 groups: control group (α-MEM) and mineralization group (α-MEM with 10^− 8^ M dexamethasone, 50 mg/L L-ascorbic acid and 10 mM β-glycerophosphate). During the culture process, the medium was replaced every three days.

### Cell transfection

For downregulation of H19, the small interfering RNA (siRNA) against H19 (si-H19) and negative control (si-NC) were designed by Genepharma (Shanghai, China). The miRNA mimics/inhibitor for miR-185-5p (mimics: 5′-UGGAGAGAAAGGCAGUUCCUGA-3′, 5′-AGGAACUGCCUUUCUCUCCAUU-3′; inhibitor: 5′-UGGAGAGAAAGGCAGUUCCUGA-3′) and negative control oligonucleotide (miR-NC: 5′-UUCUCCGAACGUGUCACGUTT-3′, 5′-ACGUGACACGUUCGGAGAATT-3′; inhibitor-NC: 5′-CAGUACUUUUGUGUAGUACAA-3′) were obtained from RiboBio (Guangzhou, China). At approximately 60% cell confluence, transfection assay was performed using Lipofectamine 3000 (Invitrogen, Carlsbad, CA, USA) according to the manufacturer’s instructions.

### RNA extraction and qPCR

qPCR assay were performed as described before [29]. Briefly, total RNA was extracted using Trizol® (Invitrogen, USA) after cell treatment. ImProm-II Reverse Transcription System (Promega, USA) was used to generate First-strand cDNA. Real-time qPCR of the reverse transcription products were determined using Permix Ex Taq (Takara, Japan) and gene-specific primers were used for qPCR in an ABI 7500HT real time PCR system (Applied Biosystems, USA). The relative expression levels of RNAs were normalized with GAPDH or U6 and calculated using the comparative 2^-ΔΔCT^ method. All experiments were performed at least three times. The primers used for qPCR were list as follow:

GAPDH-Forward: 5′-AGCCCAAGATGCCCTTCAGT-3′,

GAPDH-Reverse: 5′-CCGTGTTCCTACCCCCAATG-3′;

H19-Forward: 5′-AAGAGCTCGGACTGGAGACT-3′,

H19-Reverse: 5′-AAGAAGGCTGGATGACTGCC-3′;

miR-185-5p-Forward: 5′-CGCTGGAGAGAAAGGCAGT-3′,

miR-185-5p-Reverse: 5′-GTGCAGGGTCCGAGGT-3′;

U6-Forward: 5′-CTCGCTTCGGCAGCACA-3′,

U6-Reverse: 5′-AACGCTTCACGAATTTGCGT-3′;

IGF1-Forward: 5′-CTCTTCTACCTGGCGCTCTG-3′,

IGF1-Reverse: 5′-GCAACACTCATCCACAATGC-3′;

Osteocalcin (OCN)-Forward: 5′-AAGCAGGAGGGCAATAAGGT-3′,

OCN-Reverse: 5′-TAGGCGGTCTTCAAGCCATA-3′;

ALP-Forward: 5′-AACCCAGACACAAGCATTCC-3′,

ALP-Reverse: 5′-CCAGCAAGAAGAAGCCTTTG-3′;

Collagen I-Forward: 5′-CCCAGCCGCAAAGAGTCTAC-3′,

Collagen I-Reverse: 5′-AGCATACCTCGGGTTTCCAC-3′.

### Western blot

We used the methodology previously described by Wang et al. [29], and the membranes were incubated with primary antibodies: GAPDH (1:2000, CST, USA), OCN (1:1000, abcam, USA), ALP (1:2000, abcam, USA), and Collagen I (1:1000, abcam, USA) overnight at 4 °C. Then membranes were incubated with secondary antibody (1:5000, proteintech, USA) for 1 h at room temperature followed by detection using chemiluminescence substance (Thermo Scientific, USA). Quantity One software (Bio-Rad Laboratories, Inc., USA) was used to quantified the proteins.

### Bone-specific ALP (BALP) and collagen I protein ELISA analysis

BALP and Collagen I levels in cell medium were detected by ELISA assay using BALP ELISA kit (EM0867, FineTest, China) and Collagen I ELISA kit (EM0939, FineTest, China) according to the manufacturer’s instructions. In brief, the samples were added into the wells precoated ELISA plates followed by incubation with antibody solution at 37 °C. After three times washion, colour rendering, and termination reaction, the absorbance value of each well was detected. Last, the contents were calculated from standard curve fitted by absorbance value of standard wells.

### ALP staining

In order to assess ALP activity, the cells were prepared and washed with PBS. Then, the fixed cells were staining by ALP measurement kit (GeFan biotechnology, China). Cells were observed and photographed by a light microscope (Leica DMIRB, Germany). The staining results were confirmed by three repeated tests.

### Alizarin red staining

The mineralized nodule formation was detected by Alizarin Red staining as previously described [29]. After staining, the areas or number of positively stained mineral nodules in 3 randomly selected visual fields were measured and analyzed with Image Pro-Plus 6.0 software (Media Cybernetics, USA). Staining for each group was replicated three times.

### Luciferase reporter assay

StarBase was used to predict the interaction probability and the binding sites of H19, IGF1 and miR-185-5p. The 3′-UTR of H19 (forward primer, 5′-GACTTCTTTAAGTCCGTCTCGTTCT-3′, reverse primer, 5′-ATGACTGTAACTGTATTTATTGATG-3′) and IGF1 (forward primer, 5′-TCTATGTAAACTCTGAAAAGTAACT-3′, reverse primer, 5′-ATAAAGAAACCCTGGAGCCATAGGG-3′) were amplified by PCR. Then the amplified DNA sequences were subcloned into the pGL3-control vector (Promega, USA) immediately downstream of the stop codon of the luciferase gene. After cells were seeded into 24-well plates and grown overnight to 80-90% confluence, plasmids were transiently co-transfected with miR-185-5p mimics or miR-NC into cells using lipofectamine 3000 (Invitrogen, USA) according to the manufacturer’s manual. 48 h after transfection, the Dual-Luciferase Reporter Assay Kit (Promega, USA) was performed to determine luciferase activity.

### Statistical analysis

Data were all expressed as the mean ± standard deviation (SD) and analyzed with Prism 6.0 as described before [29]. Statistical evaluation was performed using Student’s *t* test between two groups or one-way analysis of variance (ANOVA) followed by Tukey post hoc test for multiple comparison. *P < 0.05* was considered significantly different.

## Data Availability

All data generated or analysed during this study are included in this published article.

## Abbreviations

ALP: Alkaline phosphatase
BALP: Bone-specific alp
COL1A2: Collagen type I alpha 2 chain
HCC: Hepatocellular carcinoma
HER2: Human epithelial growth factor receptor 2
HOTAIR: HOX transcript antisense RNA
IGF1: Insulin-like growth factor 1
lncRNA: Long non-coding RNA
MALAT1: Metastasis-associated lung adenocarcinoma transcript 1
OCN: Osteocalcin
